# Halophilic Carotenoids and Breast Cancer: From Salt Marshes to Biomedicine

**DOI:** 10.3390/md19110594

**Published:** 2021-10-21

**Authors:** Micaela Giani, Yoel Genaro Montoyo-Pujol, Gloria Peiró, Rosa María Martínez-Espinosa

**Affiliations:** 1 Biochemistry and Molecular Biology Division, Agrochemistry and Biochemistry Department, Faculty of Sciences, University of Alicante, Ap. 99, E-03080 Alicante, Spain; rosa.martinez@ua.es; 2 Applied Biochemistry Research Group, Multidisciplinary Institute for Environmental Studies “Ramón Margalef”, University of Alicante, Ap. 99, E-03080 Alicante, Spain; 3 Breast Cancer Research Group, Research Unit, Alicante Institute for Health and Biomedical Research (ISABIAL) Hospital General Universitario, Pintor Baeza 12, E-03010 Alicante, Spain; yoelgenaro93@hotmail.com; 4 Department of Pathology, Alicante Institute for Health and Biomedical Research (ISABIAL) Hospital General Universitario, Pintor Baeza 12, E-03010 Alicante, Spain; gloriapeiro@googlemail.com

**Keywords:** breast cancer, carotenoids, bacterioruberin, oxidative stress, antioxidant, pro-oxidant

## Abstract

Breast cancer is the leading cause of death among women worldwide. Over the years, oxidative stress has been linked to the onset and progression of cancer. In addition to the classical histological classification, breast carcinomas are classified into phenotypes according to hormone receptors (estrogen receptor—RE—/progesterone receptor—PR) and growth factor receptor (human epidermal growth factor receptor—HER2) expression. Luminal tumors (ER/PR-positive/HER2-negative) are present in older patients with a better outcome. However, patients with HER2-positive or triple-negative breast cancer (TNBC) (ER/PR/HER2-negative) subtypes still represent highly aggressive behavior, metastasis, poor prognosis, and drug resistance. Therefore, new alternative therapies have become an urgent clinical need. In recent years, anticancer agents based on natural products have been receiving huge interest. In particular, carotenoids are natural compounds present in fruits and vegetables, but algae, bacteria, and archaea also produce them. The antioxidant properties of carotenoids have been studied during the last years due to their potential in preventing and treating multiple diseases, including cancer. Although the effect of carotenoids on breast cancer during in vitro and in vivo studies is promising, clinical trials are still inconclusive. The haloarchaeal carotenoid bacterioruberin holds great promise to the future of biomedicine due to its particular structure, and antioxidant activity. However, much work remains to be performed to draw firm conclusions. This review summarizes the current knowledge on pre-clinical and clinical analysis on the use of carotenoids as chemopreventive and chemotherapeutic agents in breast cancer, highlighting the most recent results regarding the use of bacterioruberin from haloarchaea.

## 1. Introduction

Reactive nitrogen (RNS) and oxygen (ROS) species are metabolic by-products generated by all biological systems. More specifically, superoxide radicals (O_2_•^−^), hydroxyl radicals (•OH), singlet oxygen (^1^O_2_), and hydrogen peroxide (H_2_O_2_) are the most frequent ROS produced [1]. An equilibrium between ROS production and metabolization is required for most biological processes to function. When there is an imbalance in favor of ROS production, most biomolecules and cellular structures are negatively affected. Over the years, it has been repeatedly reported how oxidative stress can be one of the causes behind the onset and progression of many pathologies, including cancer, heart disease, or diabetes [2].

Cancer is considered a multi-stage process in which genetic and epigenetic alterations accumulate. These alterations produce the dominant activation of different oncogenes and the inactivation of tumor suppressor genes, ultimately leading to the malignant transformation of healthy cells [3,4]. Although a small percentage of human cancers are linked to genetic inheritance, the vast majority are caused by infections, chemical exposure and factors regarding lifestyle, such as smoking, diet, and UV radiation [5]. Over the last decades, there has been a constant rise in research focused on oxidative stress, inflammation, and cancer [6,7]. Antioxidants can counteract oxidative stress, thus helping prevent and delay in the development of this neoplasia [8]. 

Over the last years, there has been an increasing interest in microbes as natural sources for the production of carotenoids due to their remarkable antioxidant properties. The use of microbial species can be very advantageous since they produce high rates of carotenoids which can be isolated using environmentally friendly approaches; thus reducing the cost and the environmental impact compared to the chemical synthesis of carotenoids [9,10]. Extremophilic microorganisms that inhabit solar salterns (halophilic microbes) are usually exposed to high levels of oxidative stress as a consequence of high solar radiation or high temperatures (up to 50 °C in summer). In response to this stress, they have developed several molecular adaptations, such as the synthesis of carotenoids, which are very active against ROS [11]. Thus, it was described that extreme halophilic microorganisms belonging to Archaea domain (haloarchaea) can produce carotenoids, particularly rare carotenoids containing 50 carbon units, being bacterioruberin the most abundant. Haloarchaeal C_50_ carotenoids have caught the attention of many researchers due to their particular structures, which would provide them with higher scavenger activity than their C_40_ counterparts [12]. However, the actual beneficial effect of these natural antioxidants on human health is yet to be determined.

In this review, we summarize the recent advance in the use of carotenoids in preventing and treating breast cancer, highlighting the potential of bacterioruberin. 

## 2. Breast Cancer Epidemiology

Breast cancer is one of the most frequent malignancies worldwide, representing 11.7% of all cancers [13]. This neoplasia is considered genetically and clinically heterogeneous, including various subtypes, with distinct histopathological patterns and molecular characteristics, resulting in different responses to therapies and prognosis [14,15,16]. Although mortality risk decreases every year in developed countries, breast cancer incidence increases [13]. Even though there are differences between countries, it is still the leading cause of death in women between 20 and 50 years [17]. However, only less than 10% of breast cancers are thought to be hereditary. Most cases are associated with lifestyle choices, dietary habits, and environmental and reproductive factors that increase the risk of breast cancer and other chronic diseases [18,19]. Significant efforts are currently being made to develop new and improved detection strategies, therapeutic targets, and better treatments. About two decades ago, Perou and colleagues proposed an “intrinsic genetic signature” made up of 496 genes [14]. This genetic signature allowed the classification of breast cancer into four molecular subtypes, representing different biological and clinical entities [14]. Subsequent studies have made it possible to redefine these molecular subtypes [20,21,22]. However, despite different nomenclatures and molecular subtypes, breast cancer is routinely classified by immunohistochemical methods into four well-differentiated phenotypes based on the expression of estrogen and progesterone receptors (RE/RP) and human epidermal growth factor 2 (HER2): Luminal A, Luminal B, HER2-pure, and triple negative (TNBC), the latter being the most heterogeneous [23]:

- Luminal A tumors represent 50–60% of all breast cancer cases. These tumors show ER and PR expression, but HER2 is negative. In general, patients have a good prognosis since these tumors have low histological grade and proliferation rates [24];
- Luminal B tumors are also ER/PR positive, and they can present HER2 overexpression/amplification or not, with higher proliferation rates than Luminal A tumors. In addition, these tumors progress to some extent faster than Luminal A tumors [25];
- HER2-enriched tumors express neither of the two hormone receptors (HR), and they are HER2-positive. Generally, this molecular subtype is associated with a high histological grade, and, from a clinical point of view, it is characterized by having a poor prognosis. Nevertheless, therapies targeting HER2 proteins are usually successful [26];
- TNBC express neither HR nor HER2, and, therefore, they have no specific target for treatment. However, clinically, they behave more aggressively, with higher metastasis rates to the brain and lung [27].

**Figure 1:**
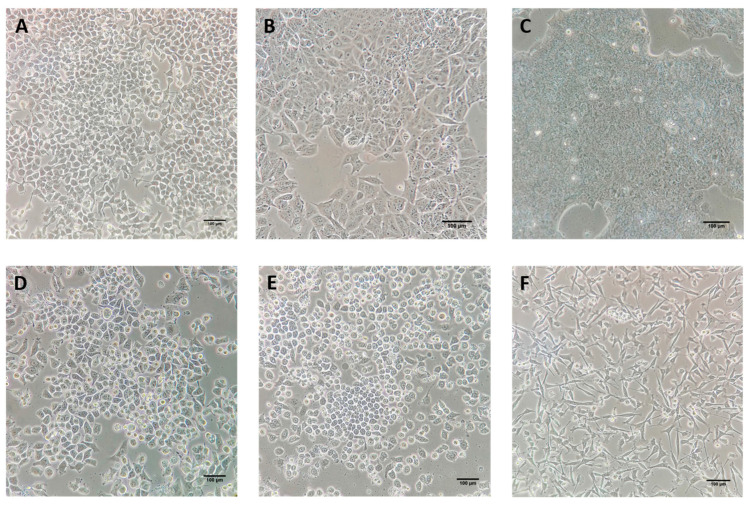
Breast cancer cell lines. (**A**) T47-D and (**B**) MCF-7 cell lines are representative of luminal A (ER/PR+) phenotypes. (**C**) BT-474 cell line represents the Luminal B/HER2+ tumors. (**D**) SK-BR-3 cell line is characterized by the lack of ER and PR expression but it overexpresses the HER2/c-erb-2 gene, thus representing HER2-enriched subtype. (**E**) MDA-MB-468 cell line belongs to the triple negative/Basal-like (ER/PR and HER2 negative) phenotype. (**F**) MDA-MB-231 cell line constitutes the triple negative/Claudin-low subtype. (Image credit: Yoel Genaro Montoyo-Pujol). Scale bars of 100 µm are included in each micrograph.

Representative cell lines for each defined breast cancer subtype are available for in vitro assays so that the distinctive effect of antitumor agents can be explored (Figure 1). T47-D (Figure 1A) and MCF-7 (Figure 1B) cell lines present an ER/PR+ phenotype, thus being examples of Luminal A subtype. BT-474 presents Luminal B features such as HER2 overexpression, as well as ER/PR expression (Figure 1C). HER2-enriched subtype can be studied thanks to SK-BR-3 (Figure 1D) and MDA-MB-453 cell lines. Among triple negative tumors, we can distinguish between triple negative/Basal-like and triple negative/Claudin low depending on gene expression characteristics [28], with MDA-MB-468 (Figure 1E) and MDA-MB-231 (Figure 1F) as their representative cell lines, respectively.

About 60–70% of breast cancers are of luminal subtype, therefore hormone-sensitive and responsive to endocrine therapy and relatively good prognosis [29]. However, HER2-positivity has been more frequently reported in HR-negative than HR-positive cancers, correlated with aggressive clinical behavior and poor prognosis. Despite the fact that novel HER2-targeted therapies have dramatically improved the outcome in HR-negative/HER2-positive patients, drug-related side effects are yet major obstacles ahead [30]. 

TNBC represents a specific subtype accounting for approximately 15–20% of breast cancers, characterized by negative ER/PR/HER2 expression. Patients show a highly aggressive clinical outcome, tending to earlier relapses and frequent metastasis to the brain and lungs, and, therefore, poorer survival compared with other subtypes [31]. 

In addition, neoplastic transformation results from the dysfunction of signal transduction networks that regulate molecular communications and cellular processes. Among them, several signaling pathways have been described to be deregulated in breast carcinoma, including the PI3K/Akt/mTOR pathway, Notch pathway, Hedgehog pathway, ERK/MAPK pathway, NF-kB pathway, FOXO1/JAK/STAT pathway, TP53 pathway, Wnt/β-catenin, as well as apoptotic and cell cycle pathways. These networks are highly adaptable and dynamic [32].

Furthermore, the results of recent retrospective and prospective clinical studies have shown that the molecular classification of breast cancer subtypes and the mechanisms of interaction between tumors and immune cells of different subtypes are significant for predicting therapeutic response and prognosis and developing individualized treatment [33]. Therefore, despite the overall successes in breast cancer therapy, which have improved the prognosis, significant challenges exist in managing and treating patients who recur, develop resistance, or show no responsiveness since they do not have therapeutic targets. Hence, it is urgent to investigate novel and more effective agents without side effects in addition to conventional chemotherapy. In this regard, carotenoids are attracting enormous attention as promising drug candidates in breast cancer treatment.

## 3. The Role of Oxidative Stress in Cancer

Cancer in humans is a multifactorial pathology triggered by endogenous and exogenous factors [34]. During the development of tumors, nutrient and oxygen concentrations change due to the dynamics of the vasculature. Combining these changes with tissue remodeling events shapes the tumor metabolic landscape, complexly involving both cell-autonomous and non-cell-autonomous mechanisms [35,36]. It is not entirely clear how tumors cope with low nutrient and oxygen concentrations. When such deficits are sensed, suitable cellular responses are elicited, and new vasculature is ultimately established [37]. Changes in mitochondrial metabolism mediate early responses to sharp drops in oxygen tension and, in particular, the generation of reactive oxygen species (ROS) [38].

Although ROS are essential in maintaining the equilibrium between pro-oxidant and antioxidant molecules, an excessive amount of these molecules negatively affects the structure and function of most biomolecules [39]. Oxidative stress can cause DNA damage and mutations, hydrolyzation of DNA bases, oncogene activation, and chromosomal abnormalities [40]. These alterations can promote tumor progression since they modify the transcriptomic profile, thus leading to impaired cell growth [41]. CpG islands can also be affected, causing loss of epigenetic information [42]. Furthermore, the oxidation of DNA by ROS releases 8-hydroxy-2-deoxyguanosine, which can generate DNA mutations [43,44]. Other possible DNA modifications include strand breaks, DNA-protein crosslinks, base-free sites, and base and sugar lesions [45]. However, not only DNA is affected by oxidative stress. ROS can oxidize lipoproteins, and the polyunsaturated lipids in the cell membrane due to lipid peroxidation [46]. In fact, lipid peroxidation is a radical chain reaction that generates cytotoxic and mutagenic compounds, such as malondialdehyde [46]. In addition, protein structure might be damaged, leading to conformational changes or loss of function [47].

ROS release during oxidative stress can be provoked by endogenous or exogenous stimuli [48]. In addition, countless enzymatic reactions in the cell are endogenous sources of oxidative stress as part of the metabolism [49]. For example, the radical O_2_•^−^ is released by lipoxygenases, cyclooxygenases, and inflammatory cells during cellular respiration [50]. However, it is well established that also lifestyle strongly influences the levels of oxidative stress, thus increasing the risk of cancer development [51,52]. 

Several oncogenic pathways are activated by high levels of ROS [53], such as the phosphoinositide 3-kinases pathway (PI3K). Phosphatase and tensin homolog (PTEN) can be inactivated by the oxidation of its regulatory Cys 124 residue due to the interaction with ROS, such as H_2_O_2_ [54]. Furthermore, the formation of a disulfide bond between Cys124 and Cys71 leads to PTEN inactivation, thus inducing the hyperactivation of the PI3K signaling pathway [55,56]. In consequence, protein kinase B (AKT) is constantly upregulated, which results in the continuous expression of genes involved in the activation of the cell cycle, for example, cyclin-dependent kinase 1 (CDK1) [57]. During the initiation of a tumor, blood vessels are poorly developed, creating a hypoxic environment [58]. Hypoxia causes an alteration in the mitochondrial electron transport chain, which releases more ROS that contributes to the activation of hypoxia-inducing factor-1 (HIF-1) [59]. More specifically, prolyl hydroxylase domain (PHD), a HIF-1 inhibitor, is inactivated in ROS. HIF-1 is a transcription factor that induces the expression of vascular endothelial growth factor (VEGF) and aerobic glycolysis [60]. In addition, tumor proliferation is enhanced due to the HIF-1-dependent activation of the c-Myc pathway [61]. High ROS levels also contribute to the invasiveness of a tumor due to the activity of transforming growth factor beta-1 (TGFß1) [62]. TGFß1 induces the epithelial-mesenchymal transition (EMT) and the secretion of various invasiveness biomarkers, such as VEGF and interleukin 6 [63]. Furthermore, ROS activates matrix metalloproteinase (MMP) synthesis via Ras and MAPK signaling pathways or via NF-kB [64]. 

Tumor cells can tolerate higher ROS levels than normal cells since they modulate the redox environment and use it to proliferate. Nevertheless, if a certain threshold of ROS levels is surpassed, even tumor cells cannot adapt, and, therefore, cell death pathways are activated [53]. 

In particular, high levels of oxidative stress in breast cancer have been reported in the literature since breast cancer cells also present an enhanced ROS production and low catalase activity. ER-positive tumors show higher levels of 8-hydroxy-2-deoxyguanosine than ER-negative [65]. Gene alterations in breast cancer are thought to be caused by ROS released by estrogen-induced oxidative stress. Breast tissue is sensitive to DNA damage by natural and synthetic estrogens [66,67]. It has been repeatedly stated that elevated ROS levels induce tumor initiation. As a consequence, cancer cells with a robust antioxidant capacity may experience selection pressure. However, cancer cells also present higher ROS concentrations than normal cells. Based on this premise, it has been suggested that cancer cells could be more sensitive than normal cells to a further increase in ROS levels, thus selectively targeting neoplastic cells [22,45]. In theory, these additional ROS would spare their effect on normal cells because ROS would be present at physiological levels [68]. However, there are still no solid results from pre-clinical and clinical studies to support this theory, and much work remains to be performed to draw firm conclusions. 

The use of antioxidants holds promises since they would exert their antioxidant activity on non-tumoral cells, whereas pro-oxidant activity would affect cancer cells. This approach is based on the pro-oxidant activity that many antioxidants presents, which will be further discussed in Section 5.1. However, pro-oxidant therapy is an emerging concept that has not been deeply explored yet. In addition, many breast cancer chemotherapeutic drugs, such as taxanes and anthracyclines, can induce oxidative stress in the brain and blood as a side effect [69].

For this reason, the administration of exogenous antioxidants has been studied during the last years to counteract the detrimental effects of neoplastic treatment in healthy tissues to prevent neurotoxicity [70]. Particularly, phytochemicals such as some carotenoids, terpenoids, and polyphenols can modulate various oncogenic pathways. Therefore, they are being investigated as potential therapeutics [71]. 

## 4. Antioxidants as a Defense Mechanism against Oxidative Stress

Antioxidants are molecules that can prevent or slow damage to cells caused by free radicals, which are unstable molecules produced during metabolic reactions, not only under “standard metabolic conditions” but also as a response to stressful environmental parameters or other pressures. They are sometimes called“free-radical scavengers”. From a functional point of view, antioxidants prevent or delay the oxidation of other molecules through the donation of hydrogen atoms or electrons. They are essential in the protection of the cells against free radicals like reactive oxygen species (ROS) and reactive nitrogen species (RNS), and, therefore, against oxidative stress [72]. 

Antioxidants can be classified into several groups based on their role, chemical composition, etc. The most used classification establishes two broad divisions, depending on whether they are soluble in water (hydrophilic) or lipids (lipophilic). Water-soluble antioxidants react with oxidants in the cell cytosol and the blood plasma, while lipid-soluble antioxidants protect cell membranes from lipid peroxidation [73]. 

Cells can use several defense mechanisms against ROS and RNS, which work together to scavenge free radicals. There are endogenous and exogenous antioxidants, the latter being synthetic or natural [74]. Cells synthesize some molecules showing antioxidant activity, such as glutathione, alpha-lipoic acid, coenzyme Q, ferritin, uric acid, bilirubin, metallothionein, L-carnitine, and small proteins such as thioredoxins (TRX). In addition, they act as an efficient reducing agent, scavenging reactive oxygen species and maintaining other proteins in their reduced state [75]. However, among the endogenous antioxidant repertoire of cells, it is worth highlighting the activity of some enzymes commonly named “antioxidant enzymes” [76]. A few of these enzymes are following listed:

- Superoxide dismutase (SOD): catalyze the breakdown of the superoxide anion into oxygen and hydrogen peroxide [77];
- Catalase (CAT): catalyze the conversion of hydrogen peroxide to water and oxygen, using either an iron or manganese cofactor [78];
- Peroxiredoxins (PRXs): peroxidases that catalyze the reduction in hydrogen peroxide, organic hydroperoxides, as well as peroxynitrite [79];
- Glutathione peroxidases (GPXs): these are enzymes involved in a more complex pathway termed “glutathione system”, which includes glutathione, glutathione reductase, glutathione peroxidases, and glutathione S-transferases. Within this series of reactions, glutathione peroxidase catalyzes the breakdown of hydrogen peroxide and organic hydroperoxides [80].

Based on the analyzed literature focused on antioxidant enzymes and cancer, the following features can be highlighted: (i) the activity of antioxidant enzymes is important for diagnosing neoplastic diseases such as non-small-cell lung cancer, bladder cancer, ovarian cancer, and colon cancer; (ii) non-small-cell lung cancer is usually characterized by decreased SOD and CAT activity and increased glutathione GST activity. Lowered SOD, CAT, and GPx activity are characteristic of bladder cancer. XOR, CAT, SOD, and GPx expression is decreased in patients with ovarian cancer. Colorectal cancer is characterized by increased MnSOD expression (in vitro studies) and SOD expression while CAT, GPx, and GR are decreased (in vivo study); and finally, (iii) SOD, CAT, and XOR are proposed as prognostic markers in cancer of the lung, bladder, ovarian, and colon [81]. Moreover, antioxidants can also be chemically synthesized, such as N-acetyl cysteine (NAC), pyruvate, selenium, butylated hydroxytoluene (BHT), butylated hydroxyanisole (BHA), and propyl gallate [82]. Some of these synthetic compounds have been tested in neoplastic cells reporting radioprotection, protection against acute toxicity of chemicals, antimutagenic activity, and antitumorigenic action [83]. However, BHT and BHA are not exempt from controversy since contradictory data involves their beneficial effects and their potentially harmful effects on human health [84]. The concerns regarding their biosafety are based on several studies reporting endocrine-disrupting effects [85], reproductive toxicity [86], and carcinogenity [87]. The controversy encourages re-evaluating the use of these synthetic antioxidants and exploring already known and new naturally derived antioxidants that may benefit human health.

Natural antioxidants are incorporated through the diet, including vitamins and carotenoids. Regarding vitamins, Vitamins C, E, and A show significant antioxidant activities. Vitamin C, also named ascorbic acid, is a redox catalyst that can reduce, and thereby neutralize ROS, such as hydrogen peroxide. Vitamin A is not a powerful antioxidant itself, but it has been reported that it plays a key role in inhibiting hepatic stellate cells (an effector of hepatocellular carcinoma) activation via suppressing thioredoxin-interacting protein and reducing oxidative stress levels. Finally, vitamin E (liposoluble) protects membranes from oxidation by reacting with lipid radicals produced in the lipid peroxidation chain reaction [88,89].

In recent decades, the relevance of antioxidants in various biological processes such as aging, cancer, and inflammation has been reported [71,90,91,92]. Different approaches have been assessed, from prevention to treatment of several pathologies. Antioxidants could also help reduce the side effects of the oxidative stress generated by chemo and radiotherapy [93,94]. Among all antioxidants, carotenoids, many of which have been identified and extracted from marine microorganisms [10,12,95], have attracted a lot of attention due to their remarkable antioxidant properties and their potential as anticancer and immunomodulatory agents.

## 5. Carotenoids

**Figure 2:**
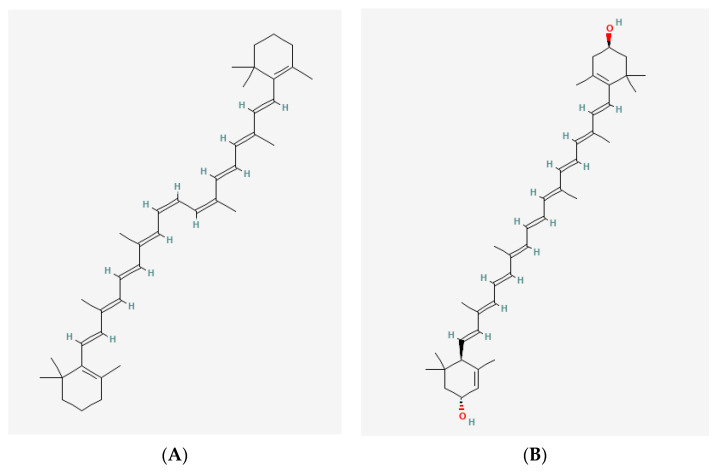
Examples of chemical 2D structures of carotenoids: (**A**) a carotenoid: cis-β,β-carotene (CID: 5927317) and (**B**) a xanthophyll: all-*trans*-lutein (CID: 6433159). The oxygen group is highlighted in red. Chemical 2D structures obtained from PubChem (NIH).

Carotenoids are isoprenoid polyenes displaying lipophilic properties. In nature, they are pigments ranging from yellow to red which can be found in plants, algae, microorganisms, and some animals [96,97]. There are more than 750 different carotenoid structures identified [98]. Carotenoids can be classified into two main groups: carotenes and xanthophylls. On the one hand, carotenes, such as β-carotene, have a chemical structure composed uniquely of carbon and hydrogen and are all vitamin A precursors (Figure 2A).

On the other hand, xanthophylls present at least one oxygen group in their hydrocarbon chain (Figure 2B) [99]. In contrast, they cannot act as precursors for vitamin A. Since carotenoids are composed of isoprenoid units, they usually contain numerous conjugated double bonds in their structure. This characteristic, combined with cyclic end groups in some cases, generates a series of stereoisomers that differ in their chemical and physical properties, such as solubility, stability, and light absorption [100]. When two parts of the structure linked by a double bond are on opposite sides of the plane, the carotenoid is in E-configuration. On the contrary, if both parts are on the same side of the plane it is called Z-configuration [101].

**Figure 3:**
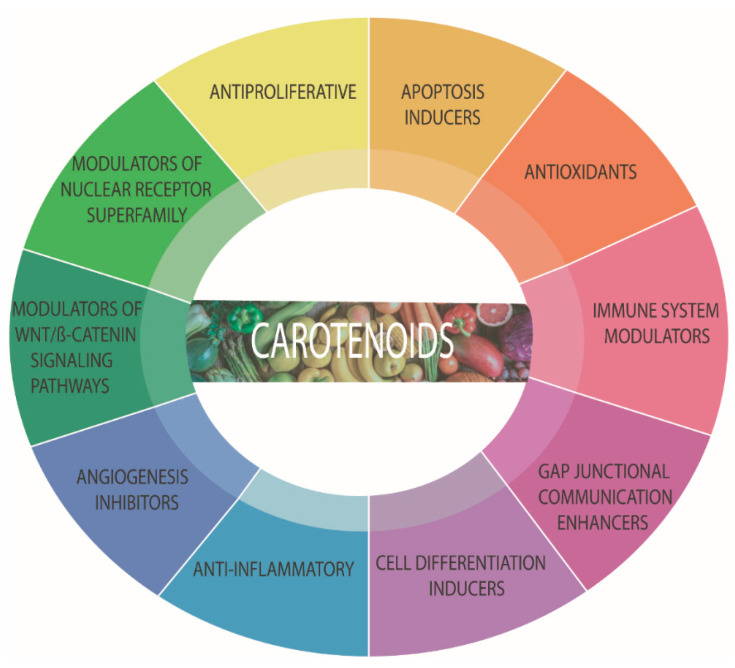
Biological properties of carotenoids. Although they are mainly known by their antioxidant activity, carotenoids can exert various effects on cells.

Fruits and vegetables contain many carotenoids, including α-carotene, β-carotene, lycopene, lutein, and zeaxanthin, among others [100]. Carotenoids are very well known for their remarkable antioxidant properties [102]. However, their relevance is not only subject to their ROS scavenging capacity. They can inhibit tumor growth and invasiveness and are apoptosis inducers, as it will be further discussed in Section 6 with the example of breast cancer [103]. Carotenoids can also modulate gene expression and possess anti-inflammatory and immunomodulatory activities [104] (Figure 3). The anti-inflammation mechanisms of carotenoids include targeting inflammatory biomarkers, such as chemokines and cytokines, a acute-phase proteins. Carotenoids can also promote PI3K/Akt and nuclear factor erythroid 2-like 2 (Nrf2) signaling pathways [105]. In addition, they can inhibit NF-kB, p38 MAPK, and JAK-2/STAT-3 signaling pathways, which are also related to tumorigenesis. Some carotenoids, such as astaxanthin, prevent neuronal death by regulating the Wnt/β-catenin signaling pathway and inducing angiogenesis [106]. However, in the case of tumor cells, carotenoids avoid the development of blood vessels, exerting an anti-angiogenic activity [107,108]. Anti-adiposity activity has also been reported for some carotenoids, such as cantaxanthin, through the differentiation of adipose cells [109]. Carotenoids have been reported to induce the proliferation of immunocompetent cells and might boost host resistance to pathogens. For example, astaxanthin positively influenced the intracellular calcium concentration and enhanced the capacity of neutrophils to eliminate microbes [102]. Furthermore, carotenoids can also increase gap junction formation, which might be related to their anti-carcinogenic properties [110].

### 5.1. Antioxidants or Pro-Oxidants?

Carotenoids’ antioxidant activity is attributed to their double-bonded structure and their ability to delocalised unpaired electrons [111]. As a result, carotenoids are capable of quenching free radicals, such as superoxide (O_2_•−), hydroxyl (•OH), and peroxyl (ROO•) radicals. Carotenoids can also prevent lipid damage from peroxidation [112]. However, recent studies have provided evidence on the pro-oxidant activity of carotenoids under certain conditions. As a consequence of this pro-oxidant potential, the concentration of ROS might increase. Nevertheless, this property does not disregard the protective role of carotenoids. Still, the conditions determining the antioxidant and pro-oxidant activity must be clarified to ensure the goal [113]. Whether a carotenoid shows pro-oxidant or antioxidant properties depends mainly on the partial pressure of dioxygen (pO_2_) and the carotenoid concentration [41]. When pO_2_ is high, a carotenoid radical is generated (Car•), reacting with O_2_ releasing a carotenoid-peroxyl radical (Car-OO•). This compound can exert pro-oxidant activity through the oxidation of unsaturated lipids [114]. In conclusion, carotenoids usually exhibit antioxidant activity in the presence of low pO_2_ whereas, antioxidant behavior is lost or becomes pro-oxidant when pO_2_ is high [115]. Elevated concentrations of a carotenoid also give rise to pro-oxidant behavior [41]. When the amount of oxidized anti-oxidant surpass certain levels, the pro-oxidant activity becomes more plausible, leading to an increase in lipid peroxidation and modulating redox-sensitive genes and transcription factors [116,117]. In addition, each type of tumor presents a particular redox status which may influence how the carotenoid interacts with ROS [118]. However, pro-oxidant activity has proven to be helpful in the treatment of some tumor cells.

## 6. Breast Cancer and Carotenoids

Among the several lifestyle factors that might contribute to cancer development, dietary habits are one of the key ones [119]. However, antioxidant compounds, such as carotenoids, present naturally in food are promising chemopreventive agents [120,121] and have chemotherapeutical properties [122,123]. Several epidemiological studies have revealed how the intake of fruit and vegetables, and more specifically of the carotenoids absorbed from these foods, correlates to a reduced incidence of different types of tumors [124,125,126]. Furthermore, carotenoids have been frequently reported to suppress the onset and progression of cancer by different mechanisms [102]. In addition, they are capable of counteracting other forms of cellular stress by modulating signaling pathways [127]. Therefore, carotenoids alone or in combination with conventional anticancer drugs might be a promising therapeutic strategy in the treatment of this pathology. Several chemotherapeutic drugs, such as alkylating agents and platinum-based compounds, release free radicals while exerting their cytotoxic activity [128]. Free radicals are partially responsible for tissue and organ injuries, such as cardiotoxicity, nephrotoxicity, and DNA damage. Although endogenous antioxidants contribute to restoring oxidative balance, these natural pigments can also quench ROS activity. For this reason, carotenoids can alleviate the side effects of chemotherapy by protecting healthy tissues with their antioxidant activity [103,129]. The supplementation of carotenoids for cancer prevention is based on several mechanisms, including a role in cell cycle progression, the Wnt/β-catenin signaling pathway, and the modulation of inflammatory cytokines [130,131,132].

### 6.1. In Vitro and In Vivo Studies

Several carotenoids have shown antitumor activity in in vitro and in vivo assays. Lycopene delayed insulin-like growth factor 1 (IGF-1)-induced cell cycle progressionand apoptosis [133,134] in the MCF-7 breast cancer cell line. Lycopene and β-carotene were confirmed to induce cell cycle arrest and apoptosis in MCF-7, MDA-MB-231, and MDA-MB-235 cell lines [135]. Although lycopene and β-carotene are classified into different groups, they have many structural similarities that suggest that lycopene could activate retinoid-like receptors. The activation of these nuclear receptors leads to the transcription of several target genes, among which we would like to highlight RARβ given that it is a tumor suppressor gene. It is worth mentioning that most breast cancer tumors and breast cancer cell lines present low levels of RARβ receptor expression, thus potentially serving as a biomarker. Carotenes can work as precursors of (all-*trans*)-retinoic acid, which acts as ligand for RAR. The mechanism of action of β-carotene might be involved with retinoic acid metabolism and the transcriptional activation of antiproliferative and pro-apoptotic genes. Another signaling pathway involved in regulating the activity of breast cancer stem cells is PI3K/Akt, since Akt downregulates glycogen synthetase kinase 3β (GSK3β) by phosphorylation in the Ser9 residue, thus stabilizing β-catenin. Wnt/β-catenin signaling pathway plays a role in modulating stem cell self-renewal, differentiation, and cell proliferation [136]. Crocin and crocetin can negatively impact the viability and the ability of invasion of triple-negative breast cancer cells (4T1) through the Wnt/β -catenin pathway [137]. β-carotene also inhibited the proliferation of MCF-7 cells by decreasing the expression of the anti-apoptotic proteins Bcl-2 and PARP and the survival protein NF-kB. It also downregulated Akt and ERK1/2, and, in consequence, there was a lower expression of superoxide dismutase-2 [122].

Recent studies have reported how lutein can induce cell death in the MCF-7 cell line while protecting normal mammary cells (SV40) from apoptosis induced by chemotherapeutical drugs [123]. Another study confirmed the antineoplastic activity of lutein by inducing apoptosis and cell-cycle arrest in MCF-7 and MDA-MB-468 cell lines. The selective effect on tumor cells seems to be due to the induction of ROS production, therefore, due to its pro-oxidant activity [138]. Mammary tumor growth was inhibited by the intake of lutein in female BALB/c mice [107]. An antiproliferative effect was also detected in fucoxanthin treatment in MDA-MB-231 cells and xenograft model [139]. Another marine carotenoid, astaxanthin, repressed cancer stem cell stemness genes and induced apoptosis in the SKBR3 cell line, indicating that it might be helpful in the improvement of current therapies [140,141]. In addition, lycopene, zeaxanthin, and capsanthin induced apoptosis in MDA-MB-231 and seem to be involved in reversing multidrug resistance [142]. Aside from those, lycophyll, luteoxanthin, and violaxanthin were also highly effective. However, lutein, antheraxanthin, and violaxanthin were moderately successful in reversing multidrug resistance.

Metastasis and cell migration can also be inhibited by carotenoids [143]. The migration of MCF-7 and MDA-MB-231 cell lines was reduced after the treatment with astaxanthin [144]. Lutein was also reported to modulate adherin, vimentin, and N-cadherin levels, which are epithelial-mesenchymal transition (EMT) associated factors [145]. In addition, it inhibited NOTCH signaling pathway which is related to cell invasion and migration [146]. Furthermore, several apocarotenoids inhibited migration and EMT associated factors in BT-549 and MDA-MB-231 [147]. Therefore, carotenoids and apocarotenoids could be helpful preventing metastasis in triple negative tumors. However, there is still lack of evidence supporting this theory and much work remains to be completed.

**Figure 4:**
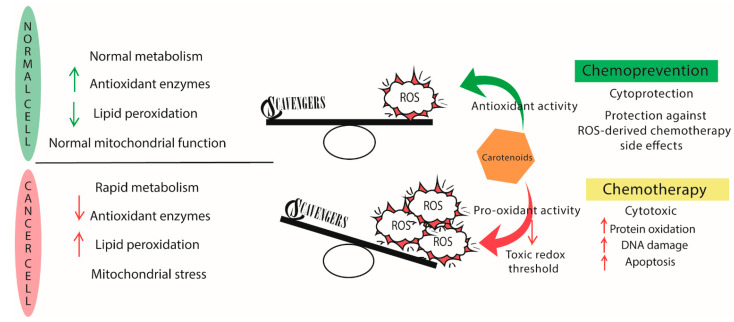
Major differences in cancer and normal cells metabolism. Over a certain ROS threshold, antioxidants present a pro-oxidant activity that leads to the apoptosis of malignant cells. Hence, its potential as chemotherapeutic agent. The antioxidant activity acts as a chemopreventive under homeostatic levels of ROS in normal cells.

Combination therapy of carotenoids with chemotherapeutic agents show a lot of promise. Recently, doxorubicin was combined with β-carotene and lutein to induce oxidative stress-mediated apoptosis in MCF-7 and MDA-MB-231 breast cancer cell lines. The pro-oxidant activity selectively affects tumor cells, sparing normal breast epithelial cells (MCF10A) [148] (Figure 4). Co-treatment of astaxanthin with the Phase I anticancer drug carbendazim showed a synergistic effect on the MCF-7 cell lines [149]. In combination with hyperthermia, crocin successfully inhibited the growth of the MDA-MB-468 TNBC cell line, whereas MCF-10A normal cells were not affected [150]. In addition, lutein and taxanes, such as paclitaxel, demonstrated a synergistic effect on MCF-7 and MDA-MB-468 cell lines [138]. Zeaxanthin and violaxanthin were capable of enhancing the antiproliferative effect of epirubicin on MCF-7 cells resistant to anthracycline [151]

### 6.2. Breast Cancer Antitumor Activity of Carotenoids: Clinical Trials

Most clinical trials start from the premise that high levels of carotenoids in plasma, obtained from carotenoid-rich foods, can prevent the development of breast cancer [152,153]. Table 1 includes all registered clinical trials which are studying the effect of carotenoids on breast cancer patients. Recent studies have associated high levels of β-carotene in plasma with lower ER-breast cancer risk [154] and with reduced systemic inflammation and cognitive improvements in breast cancer survivors [155]. It is worth highlighting the results from the trial NCT00000611, which analyzed serum concentrations of carotenoids, retinol and tocopherols in women to assess a possible association between these values and postmenopausal breast cancer risk. They concluded that indeed, high levels of α-carotene and β-carotene were inversely associated with the risk of developing breast cancer [156], which coincided with other similar studies [152]. Increased levels of carotenoids in plasma were also associated with less oxidative stress in breast cancer survivors, but inflammatory biomarkers were not affected [157]. A correlation between high levels of α-carotene and reduced breast cancer risk was found [139,158], which was consistent with the results obtained from the Nurse Health study [159] and the Women’s Health Initiative [156].

Similarly, plasma concentrations of β-carotene and β-cryptoxanthin were inversely correlated with breast cancer risk [160]. In another study, plasma total carotenoid concentration was related to a diminished risk of breast cancer recurrence in patients with an early-stage diagnosis [161]. However, not all clinical trials agree with these results. Although an association between high levels of total plasma carotenoids and reduced oxidative stress was reported in line with previous trials, these authors also concluded that carotenoids were not able to protect against breast cancer relapse in postmenopausal breast cancer survivors [162,163].

In general, most clinical trials related to carotenoids and breast cancer target the effect of carotenoid-rich food intake on breast cancer survivors [164]. However, as previously mentioned, lifestyle is critical in preventing and progressing breast cancers and the levels of oxidative stress. In this matter, oxidative stress plays a significant role in cancer development and is also deeply involved in depression, affecting how patients deal with their pathology [165]. For this reason, a recent clinical trial is assessing the effect of music therapy on different biomarkers of oxidative stress, including carotenoids (NCT04446624).

In summary, there is still not enough evidence to validate the potential benefits of carotenoids in preventing and treating breast cancer. Most clinical trials agree that a high intake of carotenoids may prevent high-risk and aggressive breast cancer, but further studies are required to draw a solid conclusion. Furthermore, no clinical trials assessing the supplementation of carotenoids in breast cancer patients, and, therefore, there is a complete lack of knowledge regarding this topic. Some studies in other types of cancer have reported controversial results [166]. Still, the chemopreventive use of carotenoids and the chemotherapeutical results in in vitro and in vivo studies encourage deepening the potential of carotenoids as part of the treatment of breast cancer patients.

## 7. Rare Carotenoids from Halophilic Microorganisms: The Future of Biomedicine?

### Bacterioruberin from Haloarchaea

Haloarchaea have been in the spotlight during the last years due to their ability to synthesize compounds of high biotechnological interest, such as bioplastics, thermophilic enzymes, and a particular type of carotenoid [12].

**Figure 5:**
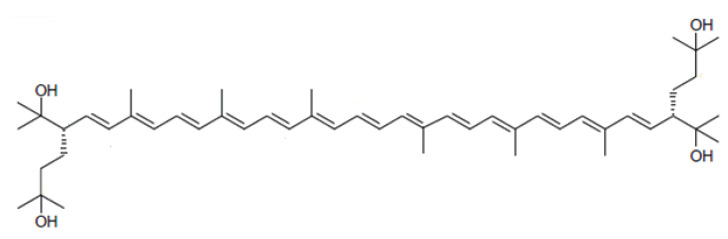
Chemical structure of the haloarchaeal carotenoid bacterioruberin.

Haloarchaea synthesize mainly a rare C_50_ carotenoid called bacterioruberin (BR) and its derivatives: bisanhydrobacterioruberin (BABR), monoanhydrobacterioruberin (MABR), and 2-isopentenyl-3,4-dehydrorhodopin (IDR) [171,172,173,174]. Other derivatives have been detected at lower concentrations, such as haloxanthin and 3,4-dehydromonoanhydrobacterioruberin; and depending on the haloarchaeal species, such as 3,4-epoxymonoanhydrobacterioruberin, which has only been described in *Haloferax volcanii* carotenoid extracts [175]. Although β-carotene, lycopene, and phytoene have also been identified in haloarchaeal extracts, they are present at low concentrations [171,176]. BR, which is the most abundant, presents an interesting chemical structure since its hydrocarbon chain is particularly long, with 50 carbon units (Figure 5). Furthermore, it possesses 13 conjugated double bonds in an all-*trans* conformation. This together with the 4 hydroxyl groups that arise from the terminal ends, provide this carotenoid with a higher scavenging potential than their C_40_ counterparts, lycopene, and β-carotene.

A recent study using *Haloferax mediterranei* describes how BR counteracts the oxidative stress generated by high concentrations of the oxidant hydrogen peroxide. BR successfully neutralized hydrogen peroxide, confirming that cells use this carotenoid to keep the oxidative balance and that this compound is indeed very efficient against ROS [176]. This distinct chemical structure has awakened the interest of many researchers during the last years due to the potential biotechnological and biomedical applications that could have [12]. Unfortunately, there is still scarce information about its antiproliferative activity. However, recent studies have reported that BR could selectively inhibit cell growth in cell lines from different cancer types, including breast cancer (MCF-7) BR induced more substantial caspase-mediated apoptosis than that of the chemotherapeutical agent, 5-fluorouracil (5-FU) and showed a higher selectivity index than 5-FU. In addition, BR was a more potent suppressor of matrix metalloprotease 9 (MMP-9) [177]. MMP-9 is one of the key proteases involved in many cancer processes, such as angiogenesis, invasion, and metastasis [178]. However, the nature of the mechanism involved is not currently clear, and therefore, much work remains to be completed. In addition, it is still unknown if it will also exert pro-oxidant activity and under what conditions. However, the successful results obtained in other biomedical areas, such as cryopreservation [179] and anti-viral activity [177] invite us to explore what BR could offer to breast cancer prevention and treatment.

## 8. Controversy and Setbacks Observed

The fact that the same molecule can exhibit antioxidant and pro-oxidant activity has been subject to controversy and has questioned the efficacy of these compounds in the treatment of tumors [118]. Another debatable point is that no consensus in the doses should be administered in clinical trials. Therefore, it is complicated to make comparisons and draw conclusions. It is also worth mentioning that endogenous factors, such as the genetic variability in antioxidant enzymes in each patient, may compromise the efficacy of these compounds [180].

Breast cancer is a very heterogeneous malignant neoplasia [181] whose different subtypes may differ in the levels of oxidative stress. The redox status of each subtype should be characterized so that the use of antioxidants, such as carotenoids, in the treatment of breast cancer can be refined. Each result contributes to a better understanding of the role of carotenoids in breast cancer patients.

However, most studies concur that consuming a collection of carotenoids is a better anticancer strategy than a high intake of one specific carotenoid. Nowadays, there is particular controversy regarding using antioxidants due to the complexity in recognizing their positive or negative effects on patient outcomes. In addition, most clinical trials have focused on the supplementation of carotenoids to diminish adverse chemotherapy effects or as chemopreventive compounds [154,164]. Although many in vitro and in vivo assays focus on the antitumor effect of carotenoids, trials focused on carotenoids as an actual treatment for breast cancer are nonexistent. Therefore, it is hard to confirm if carotenoids could be helpful in the fight against this common pathology among women. One of the potential changes in the current approach on using carotenoids in clinical trials could be intravenous administration instead of supplementation to reach a higher plasmatic concentration. What is clear is that further research on this topic is required to make a clear conclusion.

## 9. Conclusions

In closing, for many years, natural compounds have been useful in preventing many diseases. Some of those, such as taxane, was part of the development of current chemotherapeutical drugs [182]. To date, almost half of current anticancer drugs are derivatives of natural compounds or their mimics [183]. Now it is time to evaluate if carotenoids could rise from chemopreventive to chemotherapeutical agents. For this reason, preclinical research should be encouraged to elucidate what is the exact role of carotenoids in the onset and progression of breast cancer.

Moreover, the precise conditions under which a carotenoid shows antioxidant or pro-oxidant activity must be determined. Combined therapy studies are also key to establish any positive or negative interaction with current chemotherapy protocols. Finally, novel carotenoids, such as bacterioruberin, need to be investigated to deepen their potential value in treating malignant neoplasias.

## Figures and Tables

**Table 1:** Clinical trials involving carotenoids in breast cancer.

NCT Number	Status ^1^	Stage	Aim	Outcome	Reference
NCT03625635	Unknown	NA	Effect of a nutritional intervention on body composition, metabolism, and antioxidant activity	Reduced fat mass while preserving skeletal muscle mass	[167]
NCT02067481	Completed	Phase II	Effect of diet and physical activity in breast cancer survivors	Unknown	UP
NCT00000611	Completed	Phase III	Effect on higher fruit and vegetable intake on BC patients	High levels of plasma carotenoids associated with less BC risk	[156]
NCT02109068	Completed	Phase III	Effect of weight loss in BC survivors	Unknown	UP
NCT02110641	Active, no recruiting	NA	Effect of weight loss in BC survivors	Unknown	[168]
NCT04374747	Recruiting	NA	Effect of fruit and vegetable intake to reduce BC risk in lactating women	Not measured	[169]
NCT04446624	Completed	NA	Effect of music therapy in oxidative stress markers, such as carotenoids	Unknown	UP
NCT00120016	Completed	NA	Impact of a Mediterranean diet on BC risk	Plasma carotenoids increase with fruit and vegetable intake	[170]

^1^ Data obtained from ClinicalTrials.gov on 30th September 2021; BC: breast cancer NA: not applicable; UP: unpublished.
